# Glycine betaine modulates chromium (VI)-induced morpho-physiological and biochemical responses to mitigate chromium toxicity in chickpea (*Cicer arietinum* L.) cultivars

**DOI:** 10.1038/s41598-022-11869-3

**Published:** 2022-05-14

**Authors:** Deepti Singh, Chandan Kumar Singh, Dharmendra Singh, Susheel Kumar Sarkar, Saroj Kumar Prasad, Nathi Lal Sharma, Ishwar Singh

**Affiliations:** 1 Department of Botany, Meerut College, Meerut, 250001 India; 2 Division of Genetics, ICAR-Indian Agricultural Research Institute, New Delhi, 110012 India; 3 Division of Design of Experiments (DE), ICAR-Indian Agricultural Statistics Research Institute, ICAR Library Avenue, Pusa, New Delhi, 110012 India; 4 Department of Agronomy, Institute of Agricultural Sciences, Banaras Hindu University, Varanasi, 221005 India; 5 Department of Botany, Chaudhary Charan Singh University, Meerut, 250004 India

**Keywords:** Plant physiology, Plant stress responses, Abiotic

## Abstract

Chromium (Cr) accumulation in crops reduces yield. Here, we grew two chickpea cultivars, Pusa 2085 (Cr-tolerant) and Pusa Green 112 (Cr-sensitive), in hydroponic and pot conditions under different Cr treatments: 0 and 120 µM Cr and 120 µM Cr + 100 mM glycine betaine (GB). For plants grown in the hydroponic media, we evaluated root morphological attributes and plasma membrane integrity via Evans blue uptake. We also estimated H^+^-ATPase activity in the roots and leaves of both cultivars. Plants in pots under conditions similar to those of the hydroponic setup were used to measure growth traits, oxidative stress, chlorophyll contents, enzymatic activities, proline levels, and nutrient elements at the seedling stage. Traits such as Cr uptake in different plant parts after 42 days and grain yield after 140 days of growth were also evaluated. In both cultivars, plant growth traits, chlorophyll contents, enzymatic activities, nutrient contents, and grain yield were significantly reduced under Cr stress, whereas oxidative stress and proline levels were increased compared to the control levels. Further, Cr uptake was remarkably decreased in the roots and leaves of Cr-tolerant than in Cr-sensitive cultivars. Application of GB led to improved root growth and morpho-physiological attributes and reduced oxidative stress along with reduced loss in plasma membrane integrity and subsequently increase in H^+^-ATPase activity. An increment in these parameters shows that the exogenous application of GB improves the Cr stress tolerance in chickpea plants.

## Introduction

Anthropogenic activities have caused a rise in chromium (Cr) contamination of soil and water, leading to a significant reduction in crop growth, metabolic activities, and yield^1^. The major sources of Cr contamination include various industries in the agricultural ecosystem, including those involved in pigment production, electroplating, tanning, and refractory steel production as well as those producing Cr-based materials^2,3^. Plants grown in soil with high levels of accumulated metals have low yield and can potentially be toxic to humans and animals. Cr is a non-essential element in plant metabolic activities and biological processes^4–6^, and is found in several oxidative states with valency ranging from (–2 to + 6). Trivalent [Cr^+3^] and hexavalent [Cr^+6^] Cr are the most abundant stable species in nature^7^, and [Cr^+6^] is more toxic than [Cr^+3^]^8,9^. Both species differ in terms of mobility, bioavailability, and toxicity^10,11^.

When crops are grown in Cr-contaminated soils, the roots are the first to encounter heavy metals, thus beginning the processes of Cr accumulation and translocation^12,13^. Cr toxicity has many adverse effects on the morpho-physiological attributes and antioxidant defense system of plants, causing damage to root tip cells, and even plant death^14,15^. Previous studies have shown that Cr negatively influences growth, antioxidant enzyme activity, nutrient and chlorophyll content, fresh biomass, water and mineral uptake by roots and leaves, crop production and quality, and reactive oxygen species (ROS) production^4,16–19^. Furthermore, Cr stress disrupts the functions of antioxidant enzymes such as ascorbate peroxidase (APX), catalase (CAT), peroxidase (POD), and superoxide dismutase (SOD). Zhao et al.^12^ showed that Cr contamination reduced levels of manganese (Mn), iron (Fe), sodium (Na), copper (Cu), calcium (Ca), and zinc (Zn); negatively affected plant metabolic activities; and significantly reduced plant growth and crop yield. Cr toxicity damages plasma membrane functionality and induces a rapid change in the lipid composition of the cell membrane^12^. Changes in plasma membrane integrity also influence the H^+^-ATPase activity, which has been found to decrease under heavy metal stress in maize^20^, chickpea^21,22^, cucumber^23^, and rice^24^. Thus, plant characteristics that are susceptible to Cr toxicity may also be associated with plant tolerance to heavy metal contamination.

Chickpea (*Cicer arietinum* L.) the third-most produced legume crop worldwide, with an annual tons production of 17.2 million tons placing it just behind pea and bean cultivars^25–27^. A major source of protein, fats, vitamins, carbohydrates, and fiber^28^, chickpea is an excellent source of energy and is frequently used as animal fodder^29^. Studies have shown that many pulse crop species, such as chickpea^30^, mungbean^5,31^, and pea^32^, are severely affected by Cr stress. For example, Singh et al.^30^ reported the tolerance and accumulation potential of chickpea cultivars under Cr stress; hence, Cr toxicity in chickpea crops should be reduced to lower Cr accumulation in plant organs and ensure food safety. Food production technology should also be improved to mitigate and combat the effects of metal toxicity. Enhancing the ability of plants to maintain physiological activities under stressful conditions is also an effective strategy for ensuring sustainable agricultural production under conditions of fast-increasing environmental stress^33^. One such technique involves the application of glycine betaine (GB) to increase tolerance of chickpea cultivars to environmental stress, increasing crop yield. Studies on various crop species have shown that GB application under conditions of Cr contamination improves the morpho-physiological attributes and antioxidant defense system of plants^15,31,34^. However, the effects of GB on chickpea growth under Cr stress have not yet been elucidated.

The present study was designed to examine the effects of Cr stress and GB treatment on the growth of two chickpea cultivars, Pusa 2085 and Pusa Green 112. Several important morpho-physiological parameters, including (i) root morphology, plasma membrane integrity, Evans blue uptake, and leaf plasma membrane (PM) H^+^-ATPase activity were evaluated under hydroponic conditions. Meanwhile, parameters such as (ii) plant growth and biomass, chlorophyll content, oxidative stress level, antioxidant enzyme activity, proline content, ion uptake, crop yield, and Cr uptake in different chickpea plant parts were examined under pot conditions. The overall results of the present study will provide new insight regarding the underlying mechanisms of GB-induced tolerance to Cr stress. To the best of our knowledge, this is the first report investigating the application of GB to mitigate Cr stress on chickpea cultivars.

## Results

### Root morphological parameters

**Figure 1:**
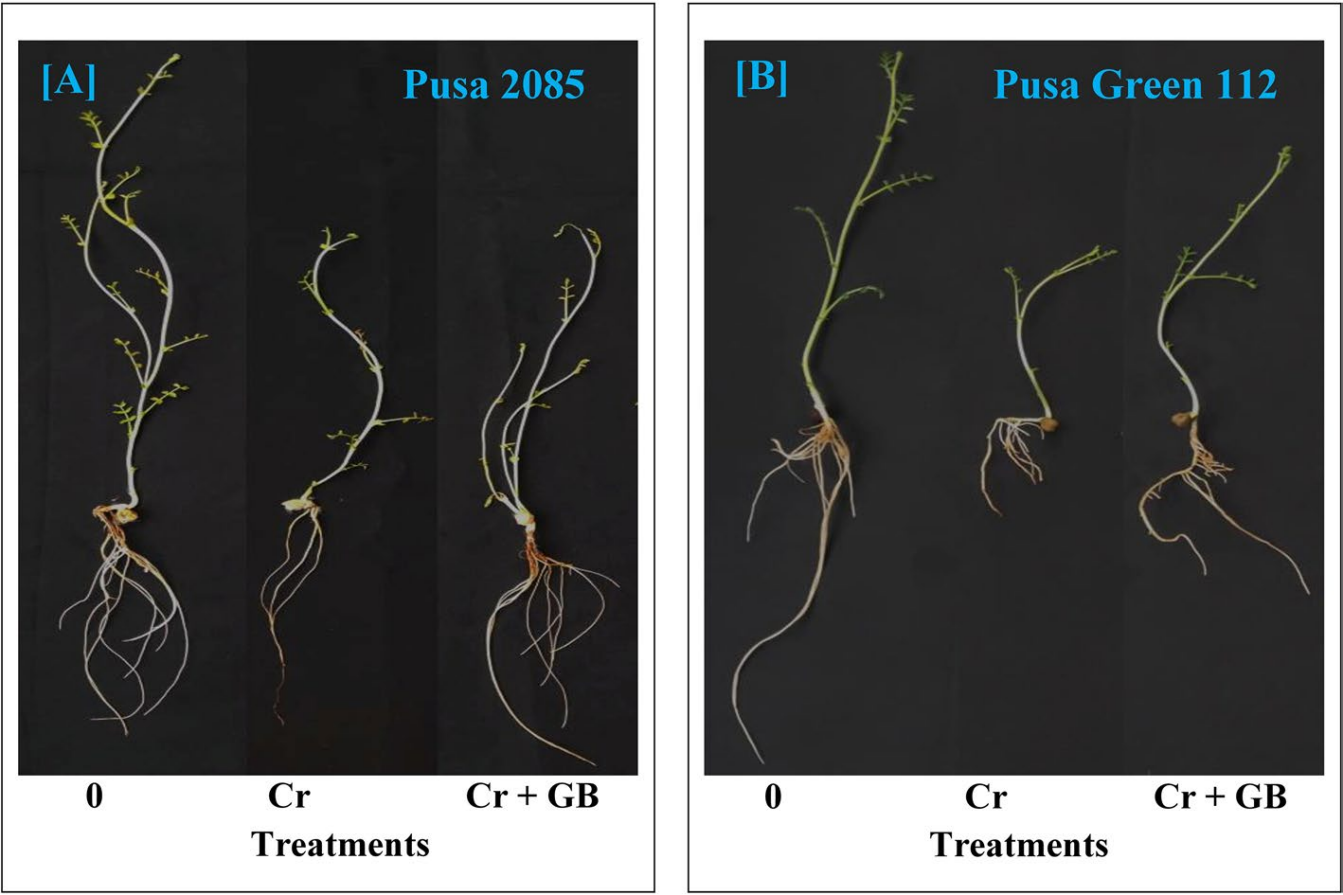
Effects of different stress levels of Glycine Betaine (GB) and Chromium (Cr), 0 and 120 μM Cr and 120 μM Cr + 100 mM (GB), on seedling growth performance of both chickpea cultivars: (**A**) Pusa 2085 and (**B**) Pusa Green 112 after 7 days of exposure (grown under hydroponics conditions).

**Figure 2:**
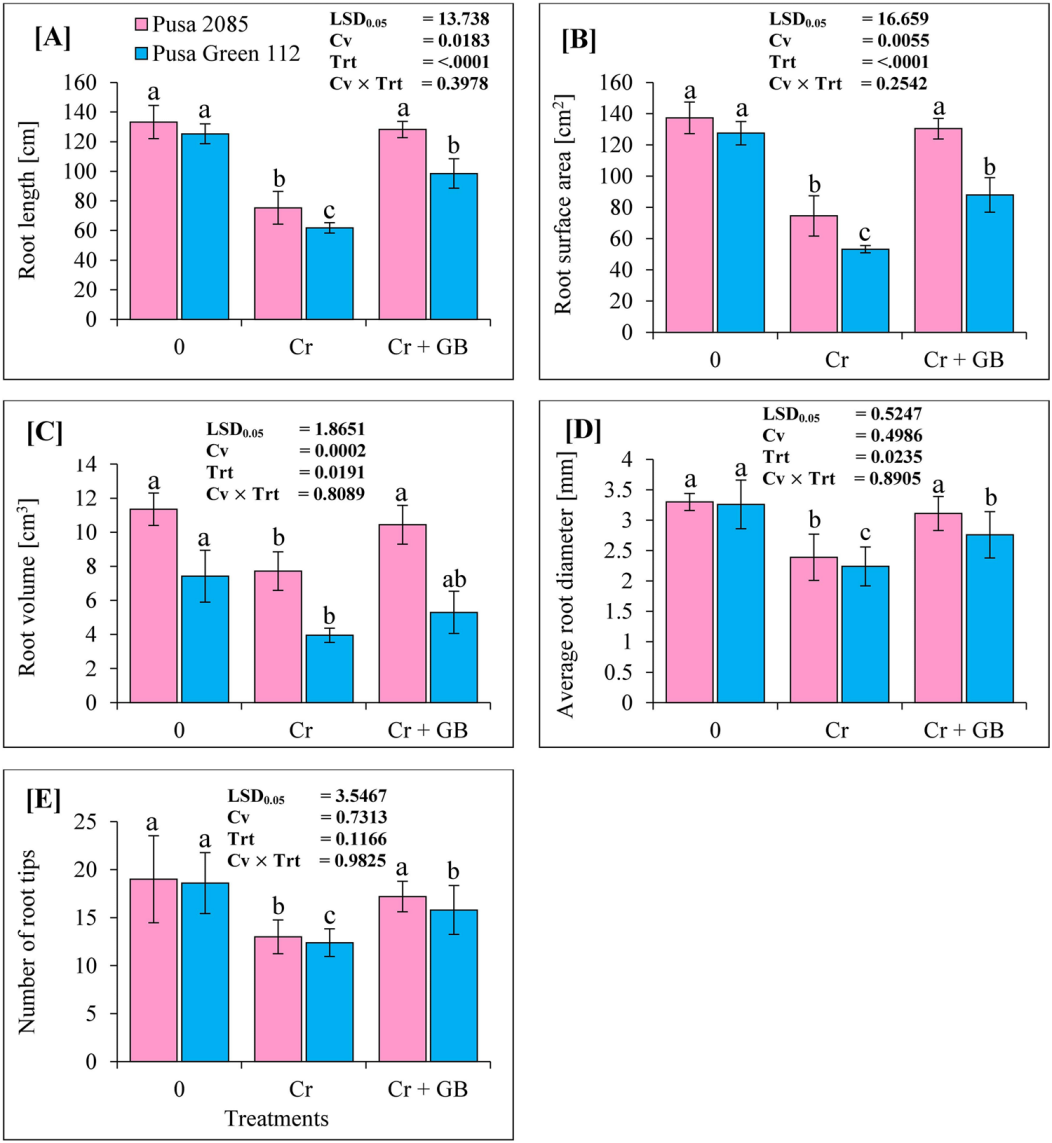
Effects of different stress levels of Glycine Betaine (GB) and Chromium (Cr), 0 and 120 μM Cr and 120 μM Cr + 100 mM (GB), on root morphological parameters: (**A**) root length (cm), (**B**) root surface area (cm^2^), (**C**) root volume (cm^3^), (**D**) average root diameter (mm), and (**E**) number of root tips of the two chickpea cultivars after 7 days of exposure (grown under hydroponics conditions). Generated data values are shown as the means of five replicates ± SE. The error bars indicated by the different letters represent significant differences, as tested by the least significant difference test (*p* < 0.05).

The effects of Cr treatment on root growth of both chickpea cultivars were evaluated via root length (RL), root volume (RV), root surface area (RSA), number of root tips (NRT), and average root diameter (ARD) measurements. Cr exposure significantly affected the root morphological parameters of both chickpea cultivars compared with those of the respective control plants (Fig. 1). Under Cr contamination, compared to the respective parameters in control plants, RL decreased by 43.46% and 50.68%, RSA by 45.72% and 58.23%, RV by 31.98% and 46.77%, NRT by 31.58% and 33.33%, and ARD decreased by 27.55% and 31.17% in the Pusa 2085 and Pusa Green 112 cultivars, respectively (Fig. 2). Notably, these parameters decreased to a higher extent in Pusa Green 112 plants than in Pusa 2085 plants (Fig. 2). However, compared to the Cr stress condition, exogenous GB treatment with Cr stress improved the root morphological parameters of both cultivars (Fig. 1). Compared to corresponding parameters in Cr-treated plants, Cr (120 µM) + GB (100 mM) treatment increased the RL by 41.23% and 37.26%, RSA by 42.82% and 39.47%, RV by 26.05% and 25.61%, NRT by 24.42% and 21.52%, and ARD by 23.02% and 18.65% in the Pusa 2085 and Pusa Green 112 cultivars, respectively (Fig. 2). These results show that roots directly responded to Cr stress by undergoing morphological changes, and that GB treatment mitigated these responses.

### Plasma membrane integrity

**Figure 3:**
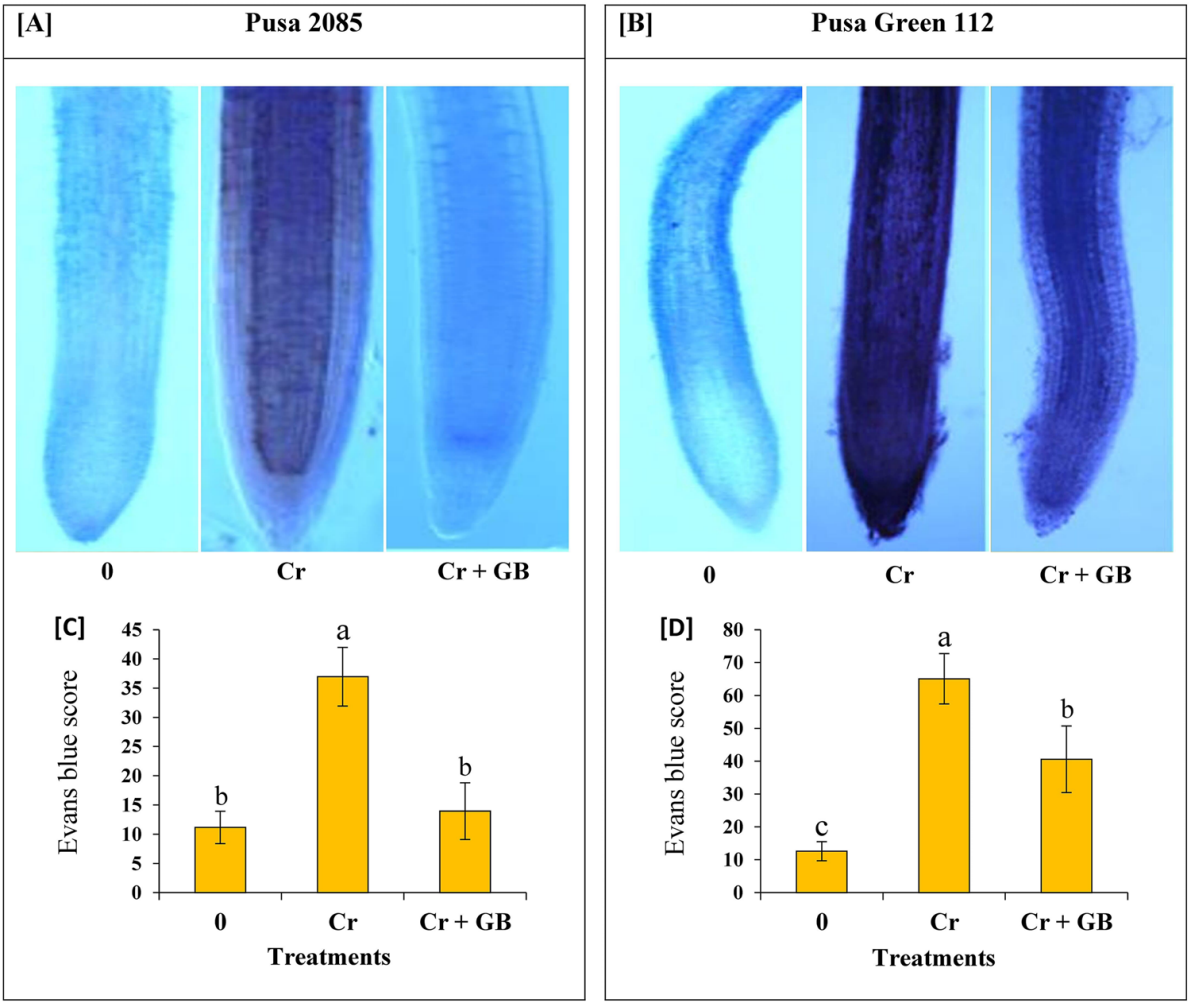
The loss of plasma membrane integrity (PMI) in the root tips of both chickpea cultivars, (**A**) Pusa 2085 and (**B**) Pusa Green 112 grown under hydroponics conditions for 7 days in the presence of different levels of glycine betaine (GB) and chromium (Cr): 0 and 120 µM Cr and 120 µM Cr + 100 mM (GB). Microscopic images of the roots from stressed and non-stressed plants stained with Evans blue. (**C,D**) Evans blue score in the root tips. The generated data values are shown as the mean of five replicates ± SE. The error bars indicated by the different letters represent significant differences, as tested by the least significant difference test (p < 0.05).

The plasma membrane integrity (PMI) in the root tips of both chickpea cultivars is shown in Fig. 3. Cr toxicity (120 µM Cr) had adverse effects on the root tip cells of both chickpea cultivars. Blue staining was much heavier in Cr-exposed plants than in control counterparts, and this heavy staining was observed in the centrally located cells in both chickpea cultivars, particularly those in the meristem region. Notably, the staining was more prominent in the cells of Pusa Green 112 plants than in those of Pusa 2085 plants (Fig. 3A,B). Exposure to Cr completely damaged the root tip cells of Pusa Green 112 plants. The root tip cells of both chickpea cultivars in the Cr + GB treatment group showed much lighter staining compared to that of the Cr-treated plants. Moreover, in the Cr + GB group, the roots of Pusa 2085 plants showed milder staining than that seen in the Pusa Green 112 roots. Quantitative estimation of the Evans Blue uptake (EBU) values in the root tips confirmed these results (Fig. 3C,D). Total EBU in the root tips of Cr-treated plants was 3.2 times (Pusa 2085) and 5.4 times (Pusa Green 112) higher than that in the control roots. After Cr + GB treatment, EBU in the root tips of the Pusa 2085 plants was reduced by a greater extent than that in the Pusa Green 112 cultivars (Fig. 3C,D). Thus, the results suggested that the Pusa 2085 cultivar was more tolerant to Cr stress than the Pusa Green 112 cultivar after GB application.

### PM H^+^-ATPase activity

**Figure 4:**
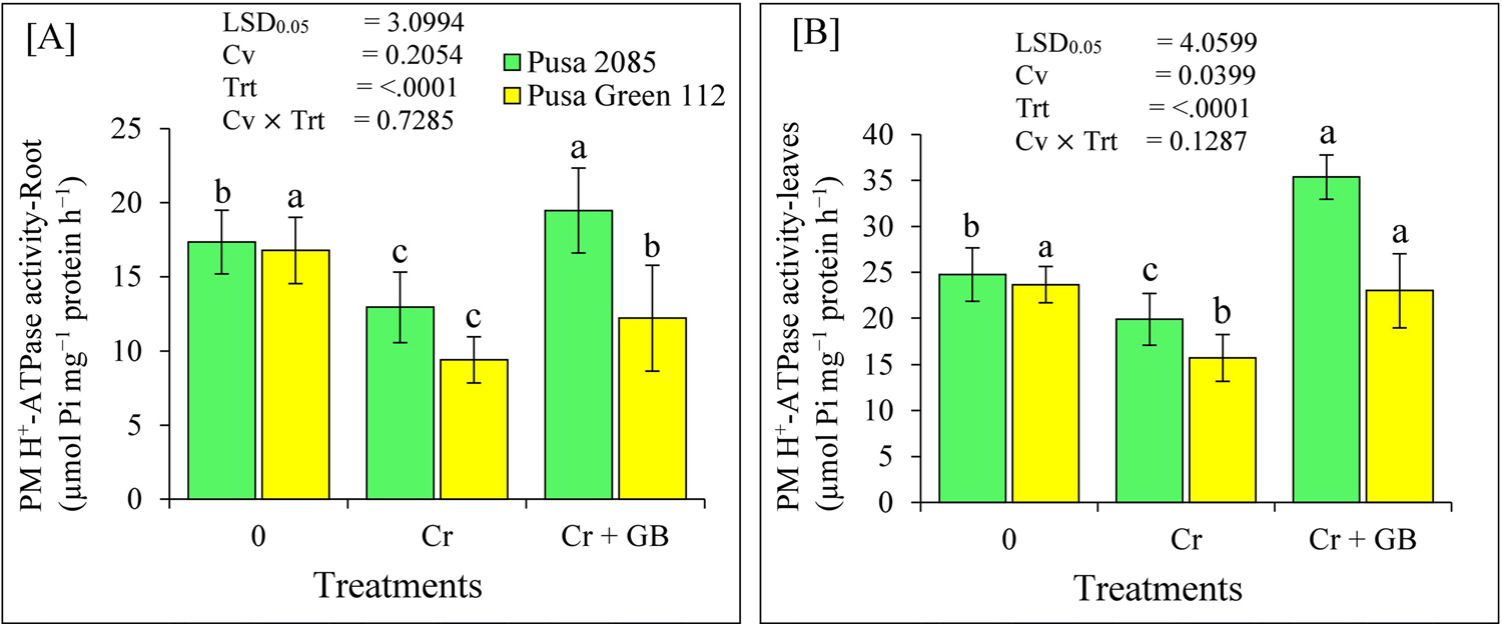
Effects of different stress levels of Glycine Betaine (GB) and Chromium (Cr), 0 and 120 μM Cr and 120 μM Cr + 100 mM (GB), on plasma membrane H^+^-ATPase activity (µmol Pi mg^−1^ protein h^−1^) in the roots and leaves of both chickpea cultivars at 7 days of exposure (grown under hydroponics conditions). Generated data values are shown as the mean of five replicates ± SE. The error bars indicated by the different letters represent significant differences, as tested by the least significant difference test (*p* < 0.05).

We investigated the involvement of GB application in PM H^+^-ATPase activities under Cr stress in roots and leaves of the chickpea cultivars. During Cr stress, plasma membrane H^+^-ATPase activities were significantly decline by 25.37% and 19.56% in the root and leaves of the Pusa 2085 cultivar, compared to untreated plants, whereas the root and leaves of Pusa Green 112 have declined by 43.89% and 35.52% (Fig. 4). However, the plasma membrane H^+^-ATPase activity increased markedly in the roots and leaves of both chickpea cultivars during Cr + GB treatment. Compared to these activities under Cr treatment, during Cr + GB treatment significant increase in the PM H^+^-ATPase activity in root and leaves of Pusa 2085 by 33.54% and 43.71% and the Pusa Green 112 in root and leaves by 22.93% and 32.68%, respectively (Fig. 4). These results showed that the PM H^+^-ATPase activity of chickpea increased significantly in both cultivars after Cr + GB treatment; however, the increase in these activities was greater in the roots and leaves of Pusa 2085 plants than in those of the Pusa Green 112 cultivar (Fig. 4).

### Morphological attributes under pot conditions

**Figure 5:**
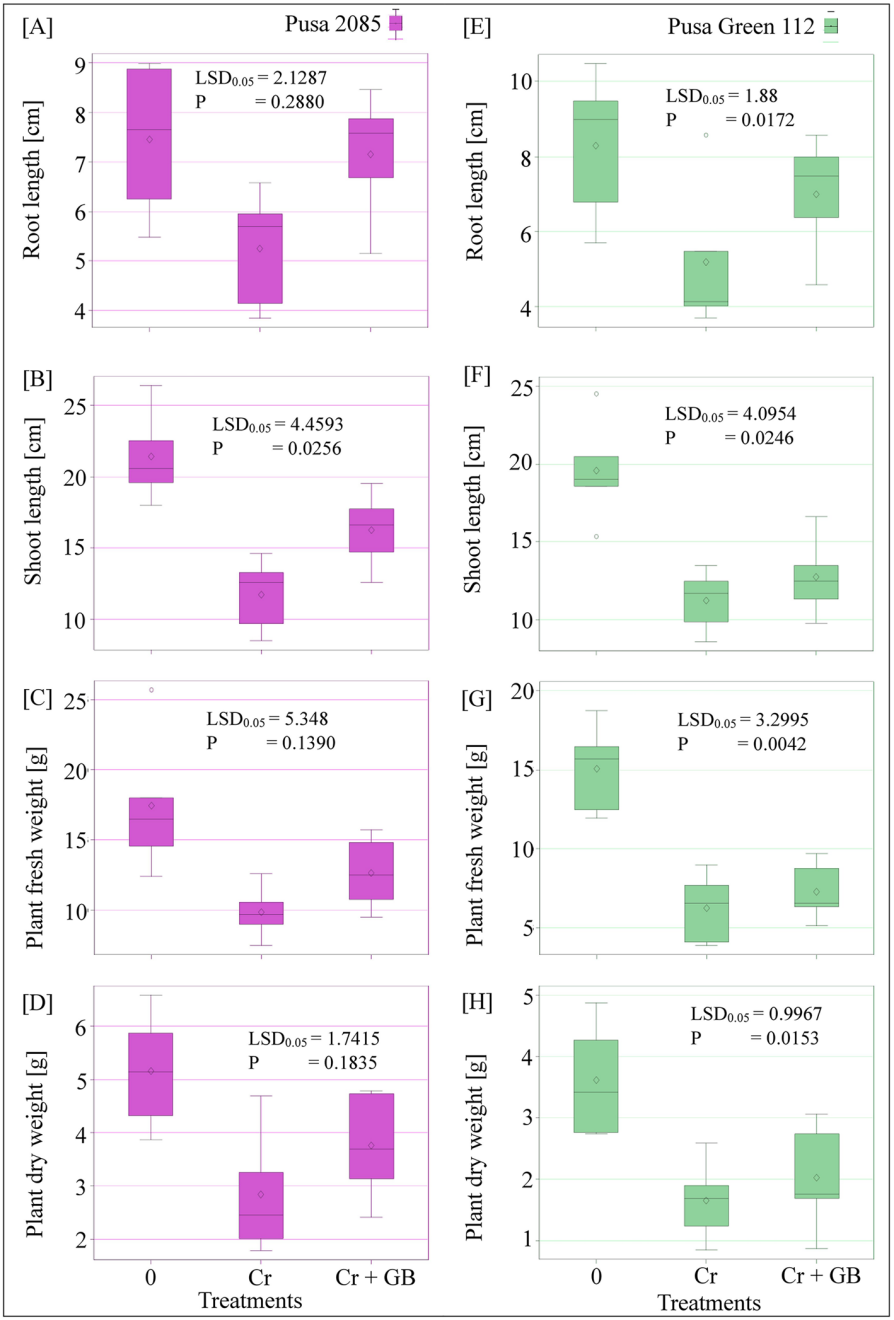
Box plot distribution showing the morphological attributes of both chickpea cultivars (Pusa 2085 and Pusa Green 112) grown (in pots) in the presence of 0 and 120 µM Cr and 120 µM Cr + 100 mM glycine betaine (GB) at 42 days after sowing. Least significant differences (LSD_0.05_) was calculated for all the treatments using SAS 9.4 software.

Compared to the morphological parameters of respective control plants, Cr treatment reduced the root length (RL), shoot length (SL), plant fresh weight (PFW), and plant dry weight (PDW) of the Pusa 2085 plants by 29.53%, 45.36%, 43.40%, and 44.96%, respectively, and of the Pusa Green 112 plants by 37.52%, 42.70%, 58.53%, and 54.29%, respectively (Fig. 5). We also observed that Pusa Green 112 plants showed a greater decline in these growth attributes than Pusa 2085 plants. Compared to the corresponding plant growth parameters under Cr treatment, Cr + GB treatment increased the RL, SL, PFW, and PDW of Pusa 2085 plants by 26.57%, 27.89%, 21.93%, and 24.47%, respectively, and of Pusa Green 112 plants by 26%, 11.86%, 14.38%, and 18.72%, respectively (Fig. 5). Thus, exogenous application of GB enhanced the growth attributes of both cultivars under Cr contamination; however, the increase in these parameters was greater in Pusa 2085 plants than in Pusa Green 112 plants (Fig. 5).

### Chlorophyll content

Significant differences in chlorophyll (Chl) component (Chl-*a*, Chl-*b*, and total Chl) content between Cr-only and Cr + GB groups were recorded in all cases (Additional File 1. Fig. S1). Chl-*a*, Chl-*b,* and total Chl were reduced in Pusa 2085 (by 58.60%, 66.13%, and 60.48%, respectively) and Pusa Green 112 (by 73.99%, 78.26%, and 75.21%, respectively) plants under Cr stress compared with values in the respective control plants. Chlorophyll contents therefore decreased by a greater degree in Pusa Green 112 plants than in Pusa 2085 plants under treatment with 120 µM Cr (Additional File 1. Fig. S1). The chlorophyll contents increased remarkedly in the leaves of both chickpea cultivars after Cr + GB treatment. Compared with the Chl levels in the leaves of Cr-treated plants, Chl-*a*, Chl-*b*, and total Chl levels increased in Pusa 2085 plants by 45%, 51.16%, and 46.45%, respectively, and in Pusa Green 112 plants by 37.50%, 31.82%, and 36.17%, respectively, under Cr + GB treatment (Additional File 1. Fig. S1). The Pusa 2085 cultivar showed greater tolerance than the Pusa Green 112 cultivar to Cr toxicity after exogenous GB application. Overall, the results showed that, under Cr stress, GB application restored chlorophyll contents in Pusa 2085 plants to a higher degree than in Pusa Green 112 plants.

### Stress biomarkers and electrolyte leakage

We measured levels of stress biomarkers, such as malondialdehyde (MDA), hydrogen peroxide (H_2_O_2_), and electrolyte leakage (EL%), in both root and leaf tissues of chickpea plants grown in soils with different Cr treatments (Table 1). Cr stress markedly increased the H_2_O_2_, MDA, and EL (%) levels in the roots of Pusa 2085 plants (by 64%, 53.24%, and 31%, respectively) and Pusa Green 112 plants (by 70.96%, 67.89%, and 50.16%, respectively), compared with corresponding levels in the control plants. Similarly, in leaves the H_2_O_2_, MDA, and EL levels increased by 59.86%, 47.61%, and 24.82% in Pusa 2085 while Pusa Green 112 showed an increase of 72.29%, 60.75%, and 41.67%, respectively. Both cultivars showed remarkable increases in H_2_O_2_, MDA, and EL (%) in the roots and leaves under Cr stress when compared to corresponding controls (Table 1). Notably, stress biomarker levels increased to a greater degree in Pusa Green 112 plants than in Pusa 2085 plants under Cr stress, indicating that Cr treatment caused more oxidative stress in Pusa Green 112 plants. Under Cr + GB treatment, H_2_O_2_, MDA, and EL (%) levels in the roots and leaves of both chickpea cultivars were reduced compared with those in Cr-treated plants without GB application (Table 1). Thus, exogenous application of GB was effective in reducing Cr-induced oxidative stress in chickpea plants.

**Table 1:** Effects of different stress levels of glycine betaine (GB) and chromium (Cr) at 0 and 120 μM Cr and 120 μM Cr + 100 mM (GB) on malondialdehyde (MDA) levels, hydrogen peroxide (H_2_O_2_) levels (μmol g^−1^ FW), electrolytic leakage (EL%), and proline levels (µmol g^−1^ FW) in the root and leaf tissues of both chickpea cultivars (Pusa 2085 and Pusa Green 112), at 42 days after sowing (grown in pots).

Cultivars	Treatments	MDA (µmol g^−1^ FW)		H_2_O_2_ (µmol g^−1^ FW)		EL (%)		Proline (µmol g^−1^ FW)	
		Root	Leaves	Root	Leaves	Root	Leaves	Root	Leaves
Pusa 2085	0	8.31 ± 0.66^b^	4.71 ± 0.60^b^	15.52 ± 1.24^b^	10.85 ± 0.92^b^	31.62 ± 3.11^c^	24.90 ± 1.90^c^	6.41 ± 0.64^b^	10.03 ± 0.71^b^
	Cr	17.77 ± 0.81^a^	8.99 ± 0.95^a^	43.11 ± 3.16^a^	27.03 ± 2.47^a^	62.62 ± 1.54^a^	49.72 ± 2.71^a^	15.27 ± 1.09^a^	37.55 ± 1.44^a^
	Cr + GB	10.25 ± 0.66^b^	5.49 ± 0.77^b^	22.11 ± 1.80^b^	14.21 ± 1.74^b^	37.46 ± 1.63^b^	32.39 ± 0.68^b^	16.31 ± 1.20^a^	41.07 ± 1.47^a^
Pusa Green 112	0	8.48 ± 0.58^c^	4.91 ± 0.49^c^	14.82 ± 0.90^c^	11.14 ± 0.79^c^	33.17 ± 0.77^c^	25.69 ± 1.55^c^	5.58 ± 0.64^b^	8.57 ± 0.66^b^
	Cr	26.41 ± 2.93^a^	12.51 ± 1.00^a^	51.04 ± 1.54^a^	40.20 ± 2.31^a^	83.33 ± 3.75^a^	67.36 ± 2.22^a^	10.65 ± 0.65^a^	20.08 ± 1.59^a^
	Cr + GB	18.99 ± 1.03^b^	8.47 ± 0.73^b^	36.12 ± 1.51^b^	31.72 ± 1.53^b^	49.95 ± 1.87^b^	44.84 ± 1.84^b^	11.19 ± 1.37^a^	21.16 ± 1.88^a^
Cultivar (Pr > F)		< .0001	0.0013	0.0001	< .0001	< .0001	< .0001	0.0004	< .0001
Treatment (Pr > F)		< .0001	< .0001	< .0001	< .0001	< .0001	< .0001	< .0001	< .0001
Cultivar × Treatment		0.0059	0.0756	0.0026	0.0002	0.0156	0.0003	0.0905	< .0001
LSD_**0.05**_		2.80	1.35	3.99	4.30	2.74	1.58	1.83	4.27

The generated data values are shown as the mean of five replicates ± SE. The error bars indicated by the different letters represent significant differences, as tested by the least significant difference test (p < 0.05).

### Metabolic antioxidant enzymes

**Figure 6:**
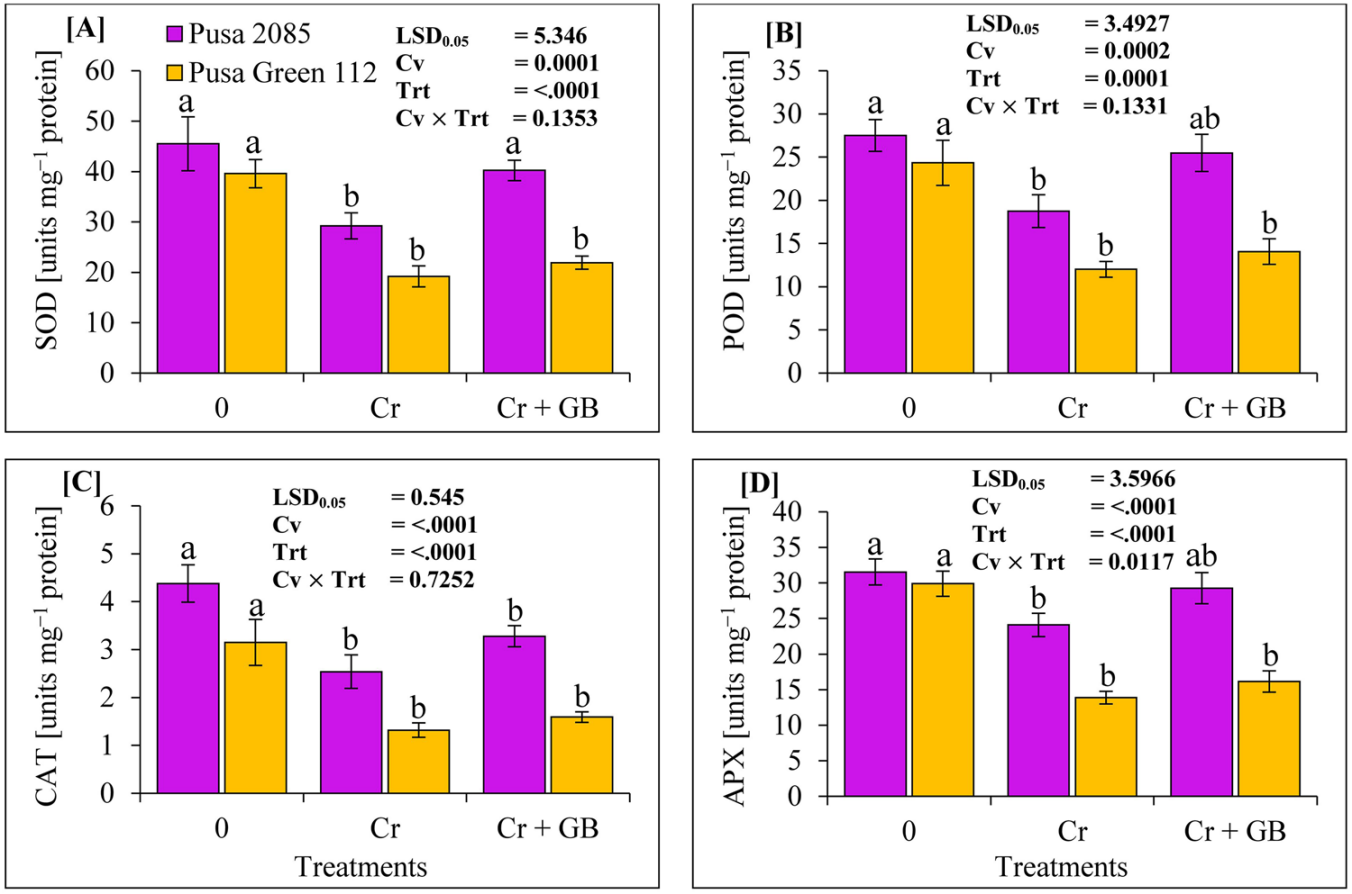
Effects of different stress levels of Glycine Betaine (GB) and Chromium (Cr), 0 and 120 μM Cr and 120 μM Cr + 100 mM (GB), on the enzymatic activities of (**A**) superoxide dismutase (SOD), (**B**) peroxidase (POD), (**C**) catalase (CAT), and (**D**) ascorbate peroxidase (APX) (units mg^−1^ protein) in the leaves of the chickpea cultivars, Pusa 2085 and Pusa Green 112, at 42 days after sowing (grown in pots). Generated data values are shown as the mean of five replicates ± SE. The error bars indicated by the different letters represent significant differences, as tested by the least significant difference test (p < 0.05).

We evaluated the activity of several antioxidant enzymes, including POD, SOD, CAT, and APX, in the leaves of chickpea plants grown under the Cr stress conditions. Compared to those in the leaves of control plants, exposure to Cr toxicity remarkably decreased the enzymatic activities of POD, SOD, CAT, and APX in Pusa 2085 (by 35.77%, 31.84%, 42.01%, and 23.61%, respectively) and Pusa Green 112 (by 51.50%, 50.65%, 58.07%, and 53.60%, respectively) leaves (Fig. 6). The enzymatic activities decreased more in Pusa Green 112 leaves than in Pusa 2085 leaves under Cr (120 µM) treatment. Compared to corresponding values in Cr-treated plants, Cr + GB treatment increased the enzymatic activities of POD, SOD, CAT, and APX in Pusa 2085 (by 27.35%, 26.44%, 22.60%, and 17.68%, respectively) and Pusa Green 112 (by 12.42%, 14.66%, 17.08%, and 14.03%, respectively) plants (Fig. 6). These results show that exogenous application of GB significantly increased the enzymatic activities in the leaves of chickpea plants under Cr stress, though the Pusa 2085 cultivar showed greater tolerance than the Pusa Green 112 cultivar.

### Proline levels

Prominent increases in proline accumulation were observed in roots and leaves of both the cultivars under Cr stress when compared to corresponding control plants (Table 1). The tolerant genotype (Pusa 2085) showed higher accumulation of proline than the sensitive one (Pusa Green 112) (Table 1). Application of GB increased proline accumulation in both cultivars, though the increase was not significant compared to that seen in the Cr-only treatment (Table 1). These findings indicate the greater tolerance of the Pusa 2085 cultivar to Cr stress under exogenous application of GB compared to that of the Pusa Green 112 cultivar.

### Nutrient elements

We measured the levels of essential mineral elements (N, P, K, Mg, and Ca) and microelements (Fe, Mn, Cu, and Zn) in the roots and leaves of both chickpea cultivars grown under different Cr treatment conditions. Under Cr treatment without GB application, significant inhibition in the accumulation of nutrient levels was noticed in the roots and leaves of both chickpea cultivars (Additional File 2. Fig. S2 and Additional File 3. Fig. S3). Although, the nutrient uptake by roots and leaves was higher in Pusa 2085 plants than in Pusa Green 112 plants under Cr treatment. However, exogenous application of GB reduced the effects of Cr stress and promoted higher nutrient uptake. Compared with the nutrient levels of Cr-treated plants, Cr + GB treatment increased the N, P, K, Mg, Ca, Fe, Mn, Cu and Zn levels in Pusa 2085 root by 34.59%, 36.69%, 36.79%, 45.45%, 40.25%, 18.64%, 35.25%, 38.30%, and 24.23%, and in leaves by 40.39%, 36.32%, 29.83%, 32.33%, 31.91%, 44.99%, 33.43%, 43.31%, and 39.89%, respectively. Similarly, Cr + GB treatment also increased nutrient uptake in Pusa Green 112 roots (by 26.10%, 23.57%, 30.63%, 28.60%, 20.83%, 22.42%, 22.19%, 20.37%, and 13.45%, respectively) and in leaves (by 21.59%, 23.06%, 17.15%, 21.82%, 18.33%, 24.22%, 21.60%, 30.52%, and 19%, respectively), when compared with Cr treated chickpea plants. Overall, the results indicated that, under Cr + GB treatment, the roots and leaves of Pusa 2085 plants exhibited higher concentrations of all nutrients than those of the Pusa Green 112 plants, suggesting that Pusa 2085 demonstrated superior tolerance to Cr stress after exogenous application of GB.

### Accumulation of Cr in plant parts

**Figure 7:**
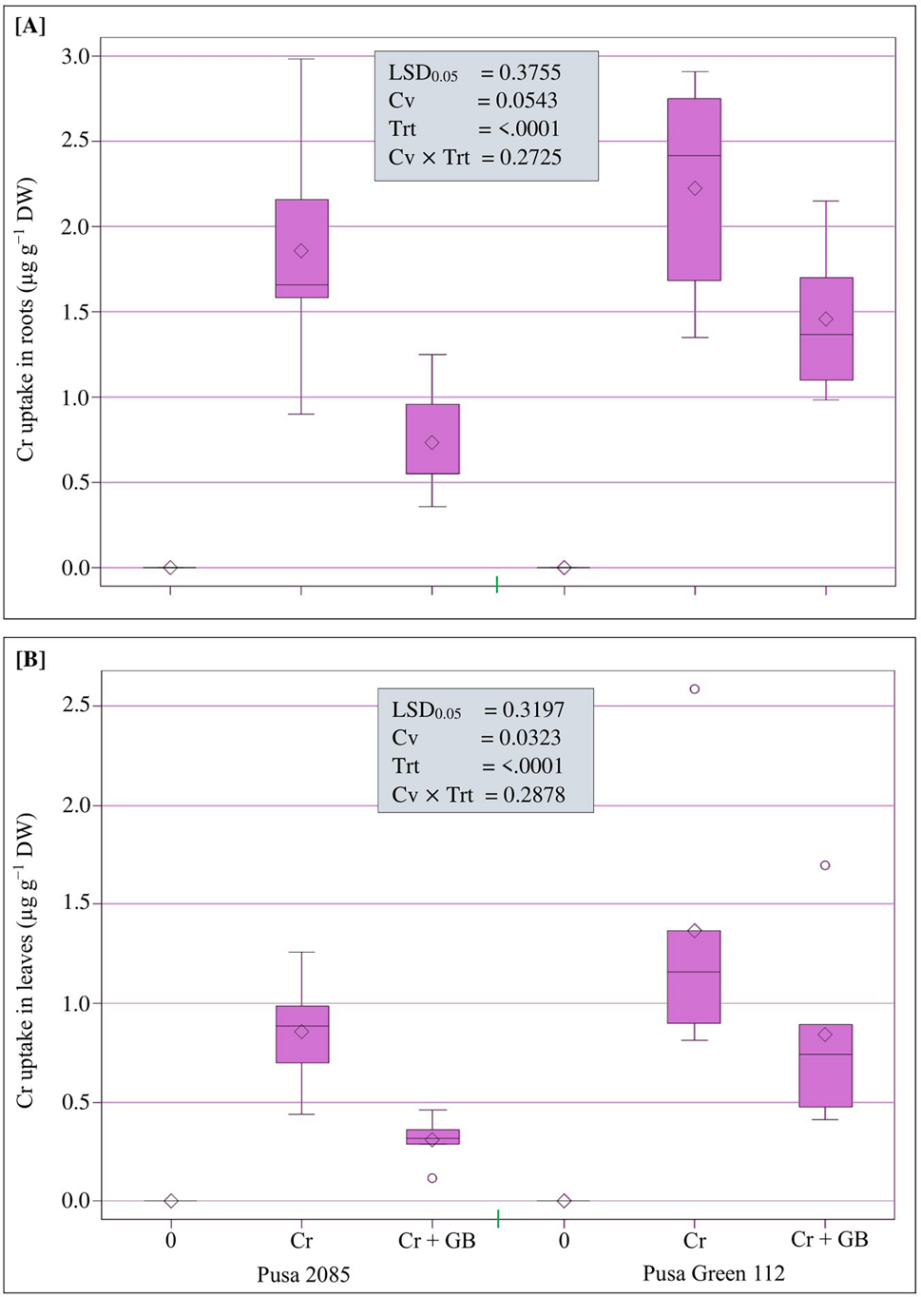
Box plot distribution of total Cr uptake (µg g^−1^ DW) in roots and leaves of both chickpea cultivars (Pusa 2085 and Pusa Green 112) grown in the presence of 0 and 120 µM Cr and 120 µM Cr + 100 mM glycine betaine (GB) at 42 days after sowing (grown in pots). Least significant differences (LSD_0.05_) were calculated for all the treatments using SAS 9.4 software.

Under Cr stress, Cr uptake was remarkably higher in roots and leaves of Pusa 2085 (1.86 and 0.85 µg g^−1^ DW, respectively) and Pusa Green 112 (2.22 and 1.37 µg g^−1^ DW, respectively) plants when compared to their respective control. Exogenous application of GB significantly reduced Cr accumulation in roots and leaves of both chickpea cultivars (Fig. 7). The roots and leaves of Pusa 2085 showed lower Cr uptake than those of Pusa Green 112 under conditions of Cr stress. However, the tissues of both cultivars exhibited reduced Cr levels under Cr + GB treatment. Moreover, the Pusa 2085 cultivar exhibited greater tolerance to Cr than Pusa Green 112 following the application of GB. In both cultivars, Cr accumulation was greater in roots than in leaves under both the conditions.

### Grain yield

**Figure 8:**
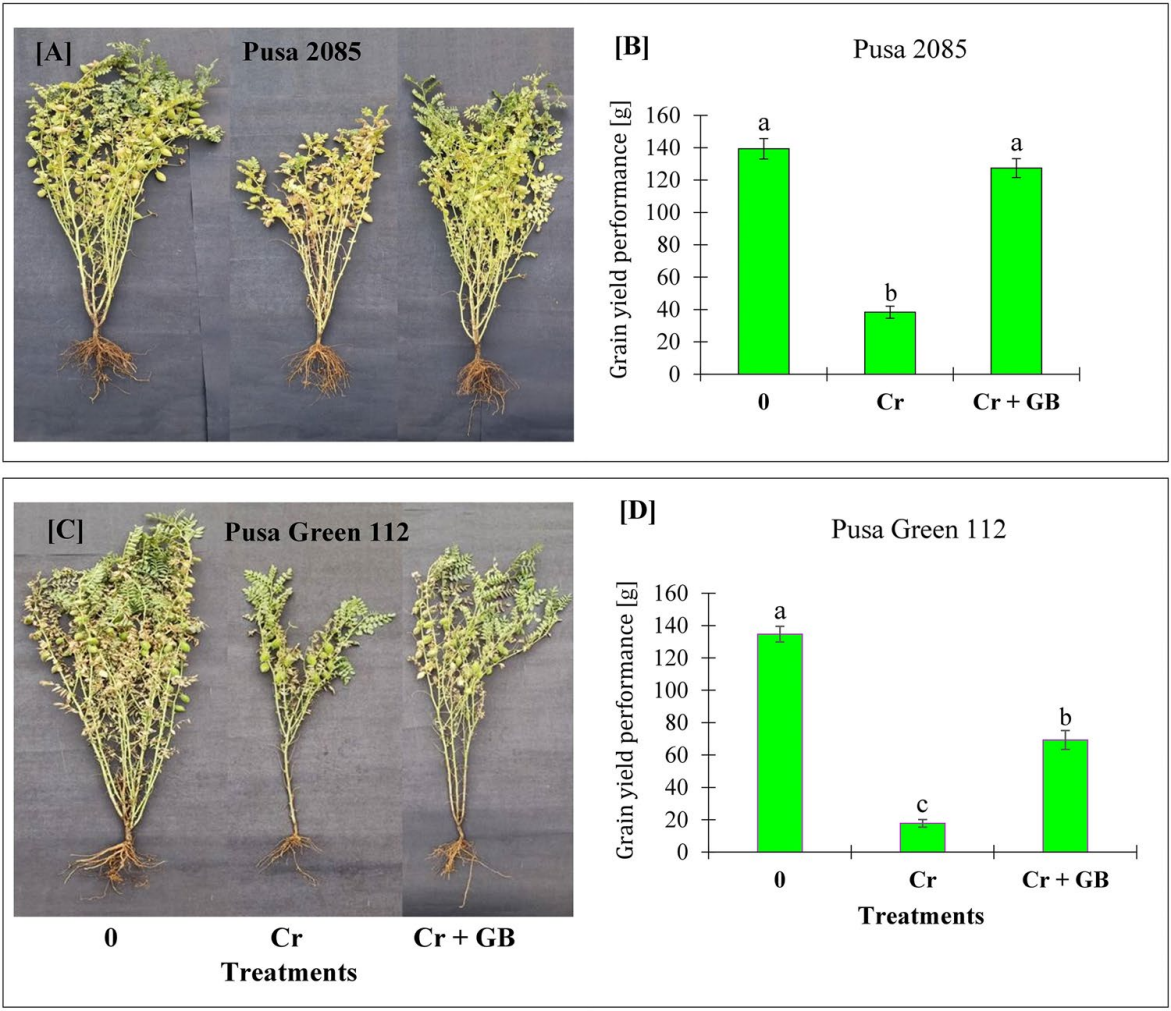
Effect of different stress levels of Glycine Betaine (GB) and Chromium (Cr), 0 and 120 μM Cr and 120 μM Cr + 100 mM (GB), on grain yield performance of both chickpea cultivars, (**A,B**) Pusa 2085 and (**C,D**) Pusa Green 112 at 140 days after sowing (grown in pots).

Compared to the grain yield of control plants, that of the Pusa 2085 and Pusa Green 112 cultivars decreased by 72.49% and 86.81%, respectively, under conditions of Cr stress without exogenous application level of GB. However, application GB significantly increased the grain yield in both cultivars (Fig. 8). Grain yield was increased by 69.91% and 67.35%, respectively, in Pusa 2085 and Pusa Green 112 plants grown under Cr + GB treatment compared to corresponding values under Cr stress (Fig. 8). Although grain yield improved in both cultivars after the application of GB, the Pusa 2085 cultivar displayed higher tolerance than the more sensitive Pusa Green 112 cultivar.

### Correlation of plant characteristics in both cultivars

**Figure 9:**
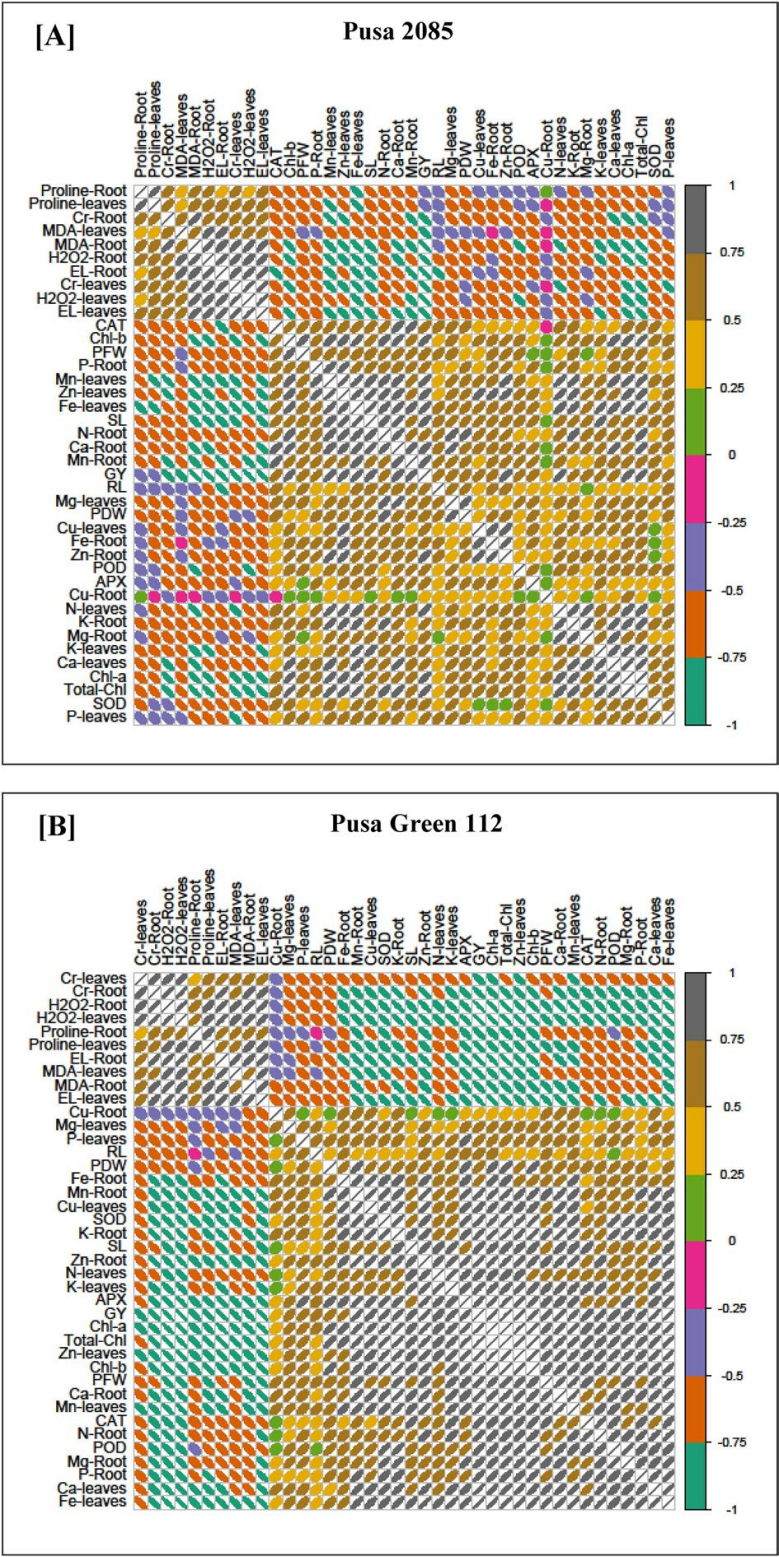
Pearson's correlation analysis between Cr uptake in the roots and leaves of the chickpea cultivars (**A**) Pusa 2085 and (**B**) Pusa Green 112 and some selected different morpho-physiological attributes, chlorophyll contents, stress biomarkers, enzyme activities, nutrient uptake, and proline contents at 42 days after sowing (grown in pots). The various attributes used in the figures are as follows: Cr-leaves, Cr-Root, H_2_O_2_-Root, H_2_O_2_-leaves, Proline-Root, Proline-leaves, EL-Root, MDA-leaves, MDA-Root, EL-leaves, Cu-Root, Mg-leaves, P-leaves, RL-Root length, PDW-Plant dry weight, Fe-Root, Mn-Root, Cu-leaves, SOD-superoxide dismutase, K-Root, SL-Shoot length, Zn-Root, N-leaves, K-leaves, APX-ascorbate peroxidase, GY-Grain yield, Chl-*a*, total chlorophyll, Zn-leaves, Chl-*b*, PFW-Plant fresh weight, Ca-Root, Mn-leaves, CAT-Catalase, N-Root, POD-Peroxidase, Mg-Root, P-Root, Ca-leaves, and Fe-leaves.

Pearson’s correlation analysis was used to identify relationships between different morpho-physiological attributes and Cr uptake in the roots and leaves of both chickpea cultivars (Fig. 9). Cr concentration levels in the roots and leaves of both chickpea plants were positively correlated with H_2_O_2_, MDA, EL, and proline levels in the respective tissues. Cr concentrations were negatively correlated with RL, SL, PFW, PDW, grain yield, mineral elements (Mg, K, Mn, Fe, N, Ca, P, Zn, and Cu), chlorophyll contents (Chl-*a*, Chl-*b*, and total Chl in the leaves), and enzymatic activities (APX, POD, SOD, and CAT) in both chickpea cultivars. These correlations indicate a close relationship between Cr accumulation and growth in chickpea plants.

### Principal component analysis (PCA)

**Figure 10:**
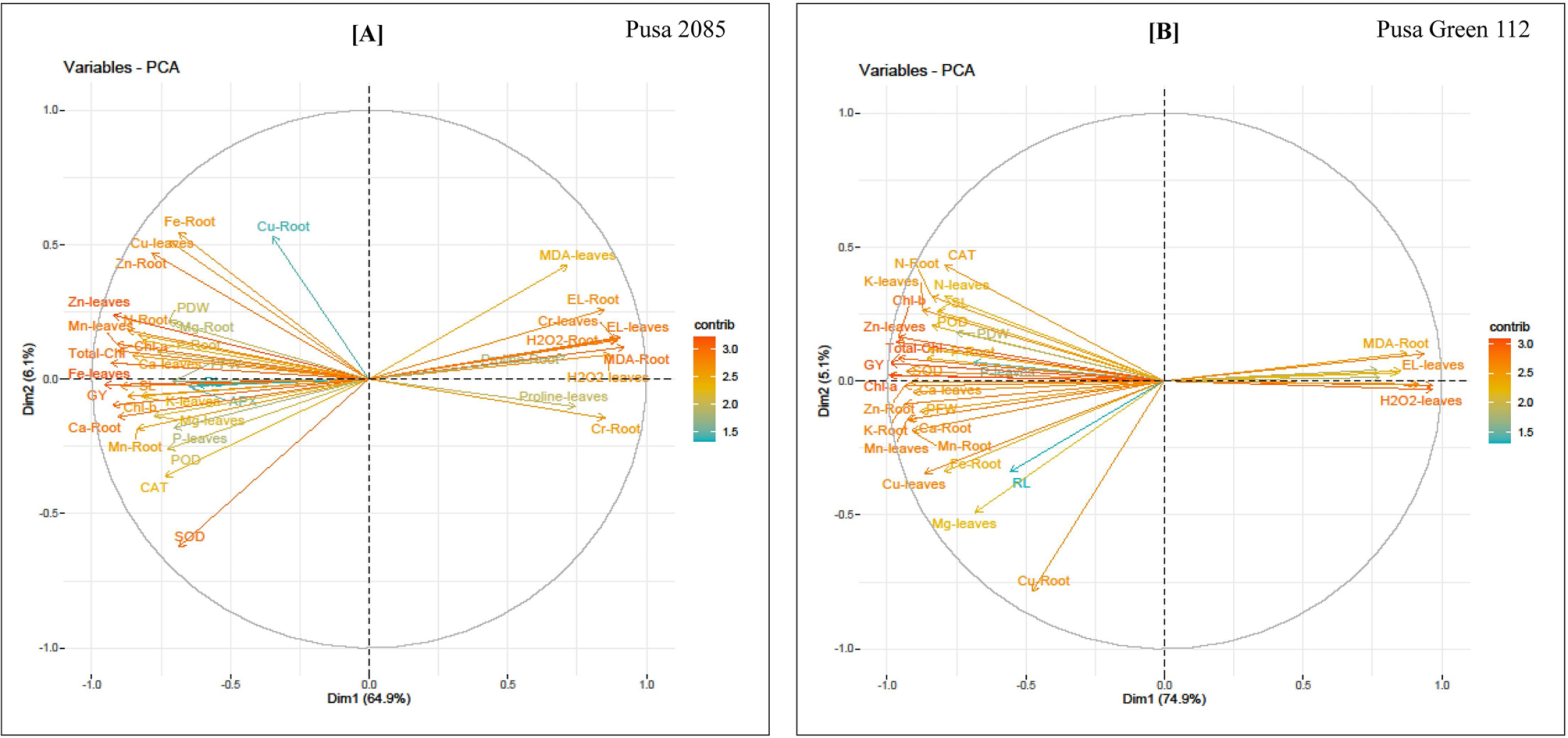
The loading plots of the principal component analysis (PCA) showing the different measured attributes of both chickpea cultivars grown in soil in the presence of 0 and 120 μM Cr and 120 μM Cr + 100 mM glycine betaine (GB). The various attributes used in the figures are as follows: Cr-leaves, Cr-Root, H_2_O_2_-Root, H_2_O_2_-leaves, Proline-Root, Proline-leaves, EL-Root, MDA-leaves, MDA-Root, EL-leaves, Cu-Root, Mg-leaves, P-leaves, RL-Root length, PDW-Plant dry weight, Fe-Root, Mn-Root, Cu-leaves, SOD-superoxide dismutase, K-Root, SL-Shoot length, Zn-Root, N-leaves, K-leaves, APX-ascorbate peroxidase, GY-Grain yield, Chl-*a*, total chlorophyll, Zn-leaves, Chl-*b*, PFW-Plant fresh weight, Ca-Root, Mn-leaves, CAT-Catalase, N-Root, POD-Peroxidase, Mg-Root, P-Root, Ca-leaves, and Fe-leaves.

PCA loading plots were prepared to measure the effects of Cr treatment on the various growth parameters of both chickpea cultivars (Fig. 10). The responses of the different parameters to Cr treatment were visualized as Dim1 (principal component 1) and Dim2 (principal component 2). Dim1 and Dim2 explained 64.9% and 6.1% of the total variance, respectively, in Pusa 2085 (Fig. 10A), and 74.9% and 5.1% of the total variance, respectively, in Pusa Green 112 (Fig. 10B). The PCA results indicated a clear separation between the cultivars with respect to growth parameters under Cr treatment (Fig. 10). Dim1 was positively correlated with MDA, EL, H_2_O_2_, proline, and Cr levels in the roots and leaves of the Pusa 2085 cultivar, and very negatively correlated with the levels of nutrient elements (N, P, K, Ca, Mg, Mn, Zn, Fe, and Cu) in both tissue roots and leaves, enzymatic activities (APX, POD, SOD, and CAT), chlorophyll content (Chl-*a*, Chl-*b*, and total Chl in the leaves), RL, SL, PFW, PDW, and grain yield (Fig. 10A). Similar results were detected in the Pusa Green 112 cultivar (Fig. 10B).

## Discussion

A variety of natural and anthropogenic activities lead to Cr contamination of water and soil, potentially leading to Cr accumulation in crops and causing serious health risks in humans and animals^35,36^. Cr has no essential metabolic functions in plants, and Cr toxicity interrupts biochemical and physiological processes, reducing crop production^,^^34,37^. Cr contamination affects the root morphological structure of plants, thereby decreasing plant growth, chlorophyll content, enzymatic antioxidant levels, plant quality, and yield. Several substances have been used in recent years to mitigate the effects of Cr toxicity on plants, including GB^15,31,34^, silicon^38^, hydrogen sulfide^4,39^, melatonin^40^ and selenium^41,42^. Herein, we investigated the effects of exogenous GB, an organic osmolyte that enhances biological and morphological processes in crops, on the growth of chickpea cultivars cultivated under Cr stress. GB was found to mitigate the effect of Cr contamination on chickpea cultivars grown under hydroponic and pot conditions.

### Effects of GB on root morphology and plasma membrane integrity in plants under Cr stress

Roots are the entry point of Cr and are the first organ to encounter the toxic effect of heavy metals. The present study revealed significant reduction in RL along with damaged ultra-structure (Fig. 1). Similar results were observed in our previous studies^6,43,44^. This could be attributed to the accumulation of Cr in root cells, or to loss of root tips cells^12,39^. Various root morphological parameters, including RL, RV, RSA, and ARD were found to be reduced in Chinese cabbage crops under Cr stress^12^, in line with the results of the present study. In addition, root morphology attributes, PMI, and ultra-structure of root tip cells were also damaged by Cr toxicity^12^. However, many studies have shown that exogenous application of GB mitigates the effects of Cr stress and improves the root growth of crop plants^3,15,34^. In the present study, application of GB reduced the effect of Cr contamination on root morphological parameters such as RL, RSA, RV, ARD, and NRT in both cultivars, however the mitigation effect was weaker in sensitive genotype (Pusa Green 112). A significant decline in these attributes was observed in both chickpea cultivars under Cr treatment, which is in line with previous findings^12,13^. GB application also improved root morphological attributes and PMI in both the cultivars when compared to those in Cr-treated plants. Furthermore, exogenous application of GB reduced Cr toxicity in chickpea plants, improving shoot growth and other physiological attributes in both cultivars. Similar results of Cr stress reduction after application of GB have been observed in mungbean^31^, wheat^3^ and cauliflower^15^.

ATPase activity is an important factor for plant survival in response to different environmental stresses, including Cr^24^, Cd^20^, and Al^45^ contamination. The basic function of PM H^+^-ATPase is to maintain the H^+^ homeostasis of plant cells and to act as the driving force for membrane transport^46^. In this study, we found that Cr affected the composition of roots and disturbed the lipid membrane, as evidenced by the increase in PMI under toxic Cr conditions. Further, the activity of the membrane enzyme H^+^-ATPase was found to be altered under Cr stress conditions. Similar results were previously found in rice roots and leaves cultivated under combined Cd and Cr exposure^24^. In the present study, ATPase activity was found to be higher in the tolerant cultivar (Pusa 2085) than in the sensitive one. A similar effect on ATPase activity has also been reported in tolerant genotypes of soybean and fava bean under Al stress^47,48^. Furthermore, the observed increment of ATPase activity seen after GB treatment suggests that ATPase activity functions to mitigate the effect of Cr ions on the plasma membrane of the plants. Therefore, high H^+^-ATPase activity can likely improve tolerance of chickpea plants to Cr stress.

In this study, chickpea plants of two cultivars showed a decline in growth parameters such as RL, SL, PFW, and PDW under Cr stress. The present study showed that exogenous application of GB alleviated Cr stress and stimulated the growth and biomass production of both cultivars, ultimately improving biological and morphological processes and crop yield. However, Pusa 2085 showed a higher degree of tolerance than the Cr-sensitive cultivar Pusa Green 112 (Fig. 5). Therefore, we showed that the Pusa 2085 cultivar has higher tolerance to Cr stress after GB application than the sensitive cultivar, Pusa Green 112. Similar results have been reported when GB was applied to sorghum plants under Cr stress^34^. Jabeen et al.^31^ reported that the exogenous application of GB significantly increased the growth of mungbean plants under Cr toxicity. Exogenous application of GB also successfully improved heavy metal stress tolerance in plants^49,50^. Many studies have also illustrated the positive effects of GB application on Cr stress in various crop plant species such as cauliflower^15^ and wheat^3^.

Cr toxicity significantly reduced the chlorophyll content in chickpea plants of both cultivars (Additional File 1, Fig. S1). Damage to the photosynthetic system is associated with Cr translocation into leaf tissue, potentially harming the laminar membrane of the chloroplast^51,52^. In this study, high Cr accumulation and translocation in plant tissues decreased chlorophyll content, which ultimately affected plant growth, physiological and biochemical traits, and yield. Both chickpea cultivars displayed a marked reduction in chl-*a*, chl-*b*, and total chl levels under Cr stress; however, this reduction was higher in the sensitive cultivar. Similar results have been reported in different crop species, including mungbean^5^, rapeseed^1^, and cauliflower^4^. GB application increases internodal elongation of shoots by increasing cell expansion and division, which may explain its effect on growth performance under Cr stress. A similar effect was previously reported in tobacco plants^53^. The improved accumulation of antioxidant enzymes and chlorophyll levels may also have contributed to the enhancement of growth performance in chickpea cultivars under Cr + GB treatment compared to that under Cr treatment alone. Previous studies have reported that the application of GB increased chlorophyll levels and photosynthesis rates in plants under heavy metal stress^50,54^. GB increases the content of photosynthetic pigments and chlorophyll, which ultimately results in enhanced plant growth and development^15^. Both chickpea cultivars showed higher accumulation of ROS and a significantly higher level of MDA and EL under Cr conditions than under control conditions. However, the damage due Cr stress was lower in tolerant genotypes than the sensitive one. Similar results have been observed in different crop plant species such as rapeseed^1^, wheat^40^, and mungbean^5^. Plants exposed to Cr exhibited high ROS production and membrane lipid peroxidation, along with increased damage to the structure and function of the cell membrane system. Similar results have also been reported in wheat^3^, mungbean^36^, and Indian mustard^41^. However, GB application reduced stress markers (i.e., ROS, MDA, and EL) in both roots and leaves of chickpea plants, indicating that GB application can effectively reduce the harmful effects of Cr toxicity (Table 1). A previous study reported that the exogenous application of GB markedly reduced the levels of these stress markers in the roots and leaves of cauliflower plants^15^. This reduction confers resistance to Cr toxicity by improving the activities of important antioxidant enzymes. These results indicate that GB application mitigates the detrimental effects of Cr toxicity and enhances plant tolerance under stressful conditions.

Both chickpea cultivars exhibited a remarkable reduction in the activities of various antioxidant enzymes (APX, POD, SOD, and CAT) and an increase in proline content under Cr stress conditions. However, there was significantly less reduction in various enzyme activity in tolerant cultivar than the sensitive one. Studies on different plant species have similarly reported a significant decrease in these antioxidant enzymes in leaves at high Cr concentrations^1,5^. However, the activity these antioxidant enzymes at lower concentrations also plays an important role in reducing Cr stress in various plant species^38,55,56^. Singh et al.^5^ reported that low Cr concentrations in nutrient solutions increased the antioxidant activity of mungbean plants, whereas high Cr levels led to a marked decrease in antioxidant activities. GB application (100 mM) enhanced the activities of antioxidant enzymes and proline in chickpea cultivars, even under Cr toxicity. Kumar et al.^34^ reported an increase in antioxidant enzymes activity and proline content in sorghum plants under Cr toxicity with GB application, which is in line with the results of present study. Proline is a basic amino acid found in proteins, and free proline plays a crucial role in plants during stressful conditions^34^. The molecular mechanisms underlying increased proline levels under Cr stress are yet to be identified, but one hypothesis suggests that protein is broken down into amino acids and converted into proline for storage. Kumar et al.^34^ reported that soil Cr increased the proline content of sorghum plants, and high Cr levels with GB application resulted in a marked increase in proline content. We also observed an increase in antioxidant activities and proline levels in plants under Cr + GB treatment, suggesting a protective role of GB against Cr toxicity. Similar findings have been reported in wheat^3^ and sorghum^34^ under Cr stress. Our results indicate that GB application is effective in ameliorating Cr stress, as evidenced by improved growth and decreased Cr accumulation in the roots of chickpea plants. Similar results were observed in mungbean^31,57^, rice^58^, and cauliflower^15^ as well.

The Cr stress also affected the levels of various nutrient elements in both chickpea cultivars. Plants grown in soil containing Cr often show deficiencies in N, P, K, and other essential nutrients because Cr can hamper root growth and cause poor performance^42^. Previous studies on Cr stress have shown that the accumulation and translocation of N, P, K, and other essential nutrients were decreased in rice^59,60^, and oilseed rape^42^. Application of GB reduces the deleterious effects of Cr by improving plant resistance mechanisms under stress conditions^61^. Under GB application, plants have many mechanisms for regulating the cellular uptake of metals and the accumulation of free metal ions ^3,62^. GB maintains essential nutrients by improving plant resistance mechanisms under stressful conditions, improving plant growth. The decrease in Cr uptake in different plant parts likely occurred due to the protective function of GB on plant cell membranes, which reduced the amount of Cr entering the cytoplasm. A similar result was previously reported by Giri^63^. Furthermore, competition between Cr and other important nutrients can reduce Cr accumulation in plants following GB application^64^. The increase in accumulation of essential mineral elements due to application of GB had a positive effect on both the chickpea cultivars. However, the nutrient levels in Pusa 2085 plants were considerably higher than those in the Pusa Green 112 plants. Similarly, GB increases the levels of important nutrients (such as Na and K ions) in the roots and shoots of wheat^65,66^. Micronutrient accumulation in plants depends on the GB application method and the plant species^3,34^. Recent research has shown that the presence of these micronutrients maintains effective plant growth, photosynthetic pigment levels, plant metabolism, and production of seeds, fruits, and carbohydrates, whereas the lack of these nutrients leads to irregular growth in plants. GB application has been shown to reduce the concentration of Cr in the roots, stems, flowers, and leaves of the cauliflower plant^15^. We measured the Cr concentration in the roots and leaves of both chickpea cultivars and showed that Cr concentrations were higher in the roots of both cultivars. Excess Cr affects the physiological and metabolic processes and yield of plants. Similar findings were also noticed by Singh et al.^5^ in mungbean plants. However, the application of GB decreased the uptake of Cr in the roots and leaves of both the chickpea cultivars. Cr uptake was lower in Pusa 2085 plants and higher in Pusa Green 112 plants. GB has also been reported to reduce Cr concentration in various plant parts^15,31^. Moreover, the foliar method of GB application has been found to be efficient in reducing Cr toxicity and improving physiological and metabolic processes in sorghum^34^.

Cr contamination of agricultural soils had negative impacts on the morpho-physiological parameters and grain yield of chickpea plants. A significant reduction in grain yield was observed under Cr toxicity in both chickpea cultivars. Singh et al.^5^ previously reported similar results in mungbean plants. However, exogenous application of GB enhanced plant growth and grain yield by alleviating the Cr(VI) stress in chickpea cultivars. GB application blocks the movement of this toxic metal, which may also explain the positive effects of GB on mungbean plants^31^. Therefore, exogenous GB can be used to improve the quality and yield of chickpea plants in Cr-affected areas. Kumar et al.^32^ reported similar improvements in grain yield after GB application under conditions of Cr stress.

## Materials and methods

### Plant materials

Two chickpea cultivars, Pusa 2085 (Cr-tolerant) and Pusa Green 112 (Cr-sensitive) were selected for use in this study based on the findings of our previous study^30^, which revealed differences in resistance to Cr toxicity. Chickpea is mainly produced in Northern India, and both these cultivars are predominant in the Hindon River region of western Uttar Pradesh. Seeds were obtained from the Pulse Research Laboratory of the India Agricultural Research Institute in New Delhi, India. All the experiments conducted in this study were performed following relevant institutional, national, and international guidelines and legislation.

### Growth conditions and root morphology attributes under hydroponic conditions

Healthy seeds of both chickpea cultivars were allowed to germinate for 14 d. The seeds were sterilized in 0.02% sodium hypochlorite (NaClO) for 5 min before germination, and then rinsed five times with distilled water. After germination, 12-day-old seedlings were transferred to plastic pots (10 L) containing half-strength Hoagland nutrient solution^67^. Before the initiation of Cr treatment, the seedlings were grown for 2 d in the growth chamber of the National Phytotron Facility (NPF), New Delhi under a dark/light period of 14/10 h. The day/night temperatures were maintained at 22/18 °C (± 2 °C), and the relative humidity was maintained at 45 ± 5%. The plants were then subjected to different Cr treatment levels: 0 (without Cr), 120 µM Cr, and 120 µM Cr + 100 mM GB. Potassium dichromate (K_2_Cr_2_O_7_) was used as the solvent. The experiment was conducted for 7 d, during which time the plants were subjected to Cr stress conditions. Each treatment included five replicates, with seven plants per replicate.

After Cr treatment, chickpea plants were harvested and separated into roots and shoots. The fresh roots were scanned using an Epson Expression automatic root scanner (1000XL 1.0, Epson America Inc., USA). Resulting root images were used to determine root morphology attributes, including RL, RV, NRT, RSA, and ARD using the WinRHIZO 200 software (Regent Instruments Inc., Canada).

### Plasma membrane integrity

Fresh root tips were treated with Schiff’s reagent followed by potassium sulfite (0.5%). The root tips were washed and then incubated with Evans Blue solution (0.025%, w/v) containing 100 µM L^−1^ CaCl2 (pH 5.8) for 30 min. PMI was determined using a light microscope (SZH-ILLD; Nikon, Japan) following the method of Yamamoto et al.^68^.

In addition to the histochemical analysis, the PMI of the root tips was quantified using spectrophotometry^69^. Immediately after harvesting, the fresh root tips of treated and non-treated plants were incubated for 30 min in Evans blue solution (0.025%, w/v) containing 100 µM L^−1^ CaCl_2_ (pH 5.8). The roots were then washed with water for 15 min, then all samples were homogenized with a 2 mL extract solution containing 50% MeOH (v/v) and 1% SDS (w/v) using a micro homogenizer. The homogenate was incubated for 15 min at 50 °C in a water bath, and then centrifuged at 10,000 × *g* for 15 min. The absorbance of the supernatant at 600 nm was determined.

### Measurement of PM H^+^-ATPase activity

The PM H^+^-ATPase activity was estimated in the fresh roots and leaves of chickpea plants following the protocol of Hejl and Koster^70^. The roots and leaves (0.5 g each) were homogenized in 1.5 mL Tris-MES buffer (12.5 mM, pH 7.8). The obtained pellet was then resuspended in 0.1 mL Tris-MES buffer (1 mM, pH 7.6). Enzyme samples (0.05 mL) were incubated in 0.2 mL Tris-MES buffer (30 mM, pH 6.5) for 20 min at 40–45 °C. Thereafter, the reaction was stopped by adding 0.16 mL SDS (10%) and incubating for 10 min at 25–30 °C. This mixture was then reacted with 0.55 mL of 0.905% sodium molybdate (Na_2_MoO_4_) and 0.04 mL 0.05% 1-amino-2-naphthol 4-sulfonic acid. H^+^-ATPase activity was quantified using a spectrophotometer (BRS, Brussel, Belgium) to estimate inorganic phosphate production at a wavelength of 700 nm.

### Growth and treatments under pot conditions

Sanitized seeds were sowed in pots filled with 6 kg of loamy soil. The pot studies were conducted at the Department of Botany at Meerut College in Meerut, Uttar Pradesh, India (77°42′47.6″ E and 28°59′29.3″ N). The initial chemical proprieties of the loamy soil are presented in Additional File 4 Table S1, as previously reported by Singh et al.^30^. Inorganic fertilizers were applied based on a previous study by Singh et al.^5^. Half-strength Hoagland solution was applied two times to each pot after 14 d intervals. After 7 d, the plants in each pot were subjected to the different treatments: 0 µM Cr (control), 120 µM Cr, and 120 µM Cr + 100 mM GB. Each treatment was repeated after a 12 d interval. The climatic conditions of the experiments were maintained at average day/night temperatures of 16 ± 6 °C and 12 ± 4 °C, respectively, and 55–65% relative humidity. The pot studies were conducted using a randomized design with seven pots per treatment. In the early vegetative stage (42 days after sowing), samples of both chickpea plants were collected to measure morphological and biochemical parameters.

### Morphological growth parameters

Chickpea plants were harvested 42 d after sowing and washed immediately with tap water and then with distilled water. Traits indicating morphological growth, including RL, SL, and PFW and PDW were measured. The fresh biomass of the plants was recorded immediately after harvesting using a digital electric balance. To determine dry biomass, the fresh plant samples of both cultivars were oven-dried at 90 °C for 12 h and at 70 °C for 6 h.

### Estimation of chlorophyll levels

Fresh leaf samples from both plants were used for chlorophyll content analysis. Chlorophyll was extracted from 0.5 g of leaf samples using an aqueous acetone solution (85%, v/v). The extract was centrifuged at 3000×*g* for 10 min at 4 °C. The supernatants were then diluted in an 85% aqueous solution at a concentration appropriate for spectrophotometric (1800UV, Shimadzu, Japan) analysis. The extinction coefficient was estimated against a blank sample (pure 85% aqueous acetone) at different wavelengths (663 and 645 nm), and the chlorophyll level was calculated using a standard method^71^.

### Determination of MDA, H_2_O_2_, and EL

After 42 days of growth, the roots and leaves of all plants were used to determine MDA, H_2_O_2_, and EL levels. MDA levels were estimated in untreated and treated chickpea plants following the method explained by Heath and Packer^72^. H_2_O_2_ levels were determined according to the protocols described by Jana and Choudhuri^73^, and EL level was measured using the procedure described by Valentovic et al.^74^. The EL percentage was calculated using the following formula: EL% = EL1/EL2 × 100.

### Analysis of metabolic antioxidant enzymes

To measure enzyme activities, 0.5 g fresh leaf samples were collected and ground in potassium phosphate (KH_2_PO_4_) buffer solution (pH 7.0) under pre-chilled conditions. Different buffer solutions were used for each enzyme. The samples were centrifuged at 10,000×*g* at 4 °C for 15 min, then the supernatant was extracted, stored in micro centrifuge tubes, and used to determine the activities of different enzymes. SOD and POD activities were analyzed based on the methodology of Zhang^75^, CAT activity was measured according to the method of Aebi^76^, and APX activity was measured according to the methods described by Nakano and Asada^77^.

### Determination of proline levels

The proline content of roots and leaves was examined following the method of Bates et al.^78^. The proline levels in the root and leaves samples were determined at 520 nm using a spectrometer (1800-UV, Shimadzu) and expressed as µmol g^−1^ FW.

### Determination of nutrient elements and Cr uptake

Samples were separated into roots and leaves, oven-dried at 90 °C for 12 h and at 70 °C for 6 h, and ground into powder. Then, 1 g samples were extracted and mixed with 5 mL of HNO_3_ and 1 mL of HCLO_4_, and the total solution (25 mL) was filtered. The levels of nutrient elements, including Mg, K, Mn, Fe, Ca, Zn, Cu, and Cr, were estimated using an atomic absorption spectrometer (AAS model ZEEnit 700P, made by Analytik Jena, Germany) following a standard method of Gill et al.^6^. Phosphorus and nitrogen levels were measured according to the methodologies of Murphy and Riley^79^ and Singh et al.^30^, respectively.

### Grain yield

Both chickpea cultivars were harvested at the mature stage, 140 days after sowing. In total, seven plants were chosen from each treatment group (with four replicates) to measure grain yield. Grain yield was measured as the weight of 100 randomly-selected grains from each group.

### Statistical analysis

Statistical analysis was conducted using SAS software (version 9.4, NC, USA), and mean values were compared using the least significant difference (LSD) test. The results for both chickpea cultivars were described mean ± standard error (SE). The differences between treatments were determined using two-way analysis of variance, and the LSD test (*p* < 0.05) was used for multiple comparisons between mean values. The figures and PCA plots of chickpea parameters were prepared using R statistical software. The Pearson correlation coefficients between the different attributes of chickpea plants were also calculated.

## Conclusions

In conclusion, our findings showed that Cr toxicity had a negative effect on plant morpho-physiological characteristics, including root morphology, PMI, biochemical parameters, and nutrient levels in both chickpea cultivars. Furthermore, Cr stress also increased oxidative stress (H_2_O_2_, MDA, and EL) and amino acid (proline) levels in the roots and leaves of both cultivars, ultimately reducing plant metabolism and yield. We explored the mechanisms by which plants mitigate Cr toxicity, as well as the effects of GB treatment on growth characteristics. The exogenous application of GB significantly enhanced plant growth and yield, along with physiological and biochemical attributes in chickpea plants. The Cr-tolerant cultivar, Pusa 2085, showed better performance under Cr stress with GB application (100 mM) than the Cr-sensitive cultivar, Pusa Green 112. Moreover, GB application reduced Cr accumulation and oxidative stress in the roots and leaves by increasing antioxidant enzyme activities and physical processes. Exogenous GB effectively reduced the Cr accumulation and improved PMI and H^+^-ATPase activity. Thus, the annual loss of crop yields due to heavy metal toxicity can be reduced with GB application. GB application also significantly increased the nutrient content in the roots and leaves of both cultivars under Cr toxicity and contributed to better growth and greater tolerance in Cr-treated plants, thereby decreasing Cr uptake and translocation. Hence, exogenous GB application can be used to improve the quality and yield of chickpea plants in Cr-contaminated areas. To our knowledge, this is the first report exploring the mechanism of action of GB in chickpea plants under Cr stress. Further studies using field trials are needed to investigate the effects and mechanism of action of GB in different crops under Cr toxicity.

## Supplementary Information

Supplementary Figure S1.

Supplementary Figure S2.

Supplementary Figure S3.

Supplementary Table S1.

Supplementary Legends.

## Data Availability

The data used to support the findings of this study are included within the article/supplementary material.
