# Supplementary figures and images for: Glycine betaine modulates chromium (VI)-induced morpho-physiological and biochemical responses to mitigate chromium toxicity in chickpea (Cicer arietinum L.) cultivars
Source: Sci Rep. 2022 May 14;12:8005. doi: 10.1038/s41598-022-11869-3 (PMC9107477; doi:10.1038/s41598-022-11869-3)

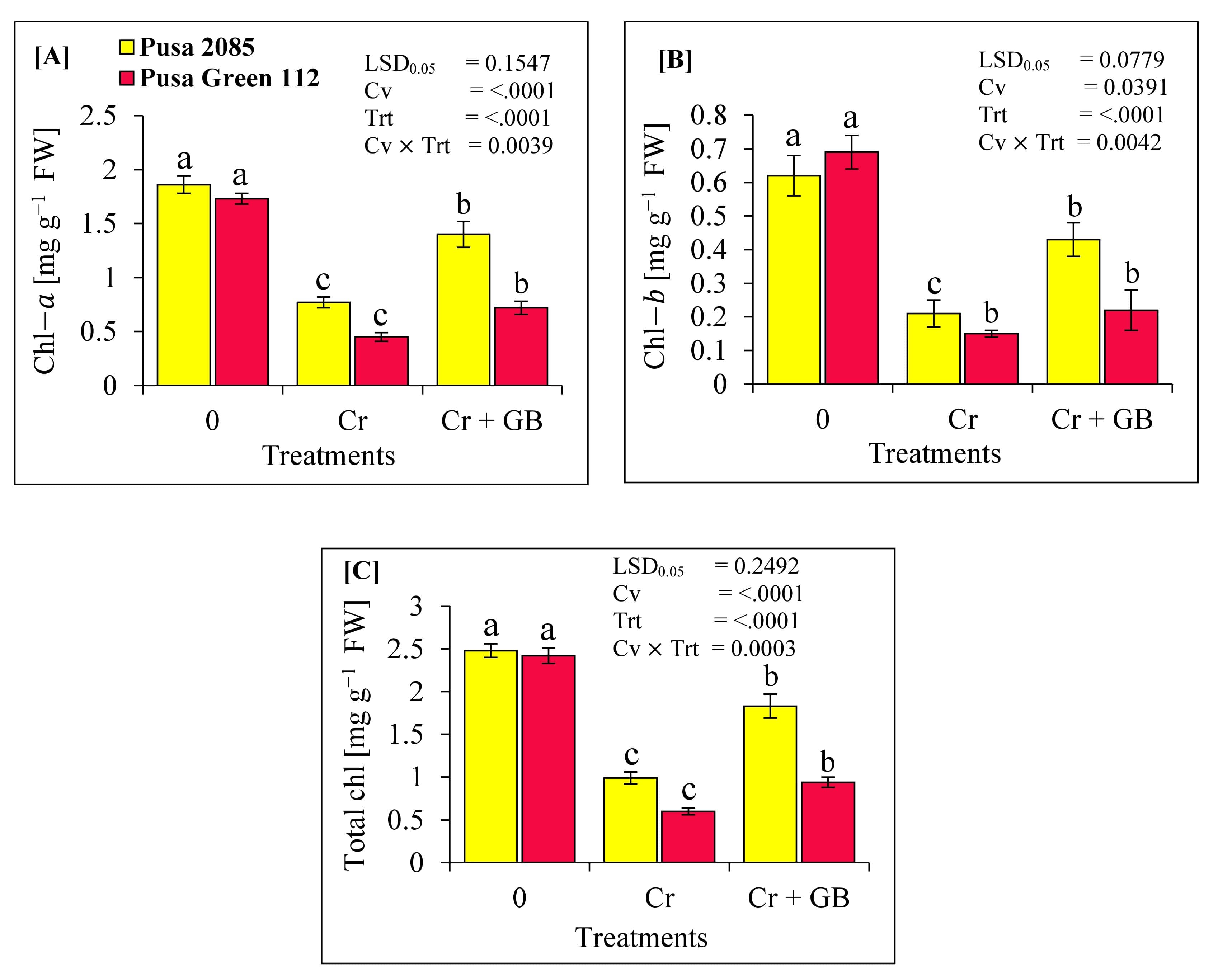

Supplement: Supplementary file 1 — Supplementary Figure S1. [file 41598_2022_11869_MOESM1_ESM.jpg]

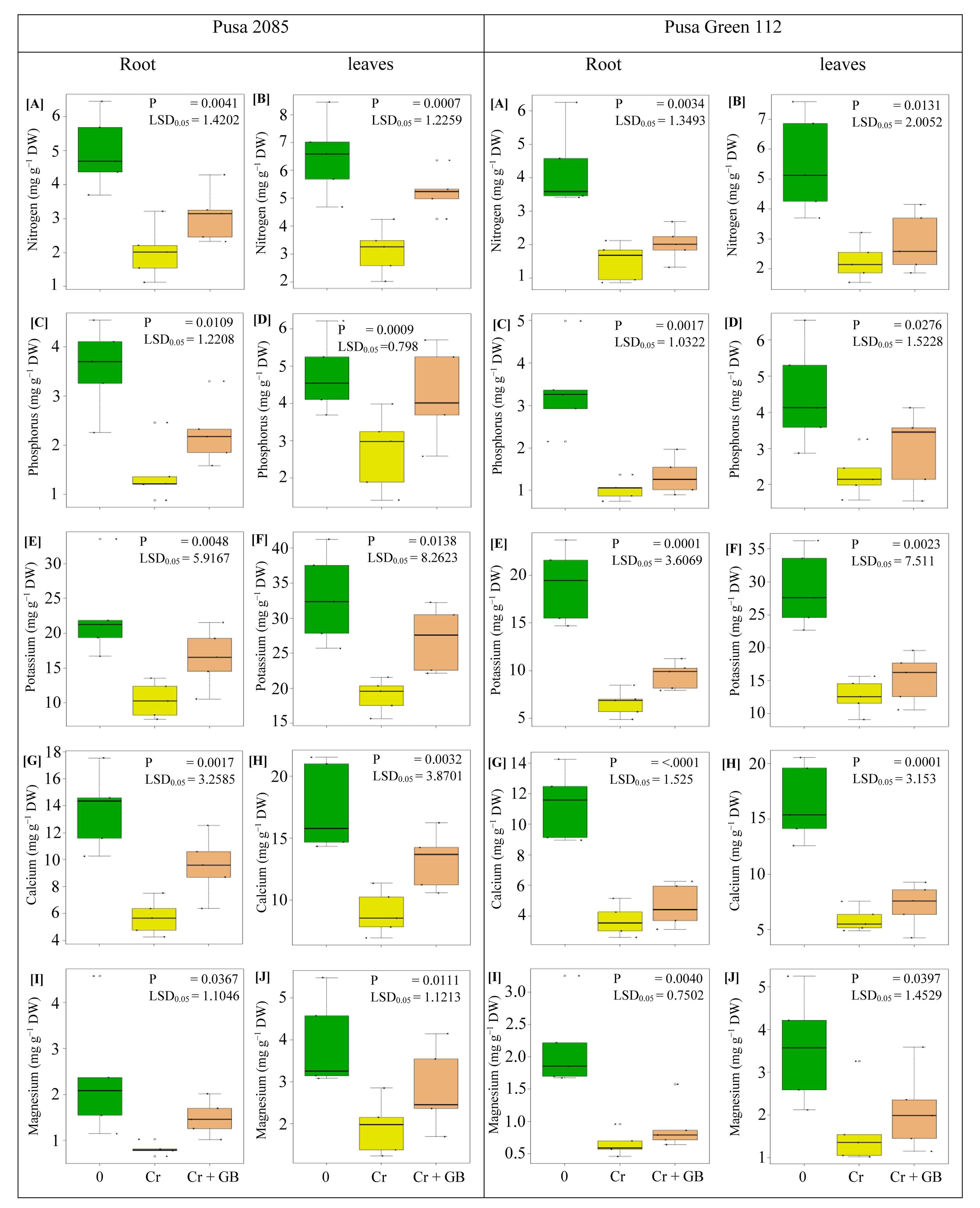

Supplement: Supplementary file 2 — Supplementary Figure S2. [file 41598_2022_11869_MOESM2_ESM.jpg]

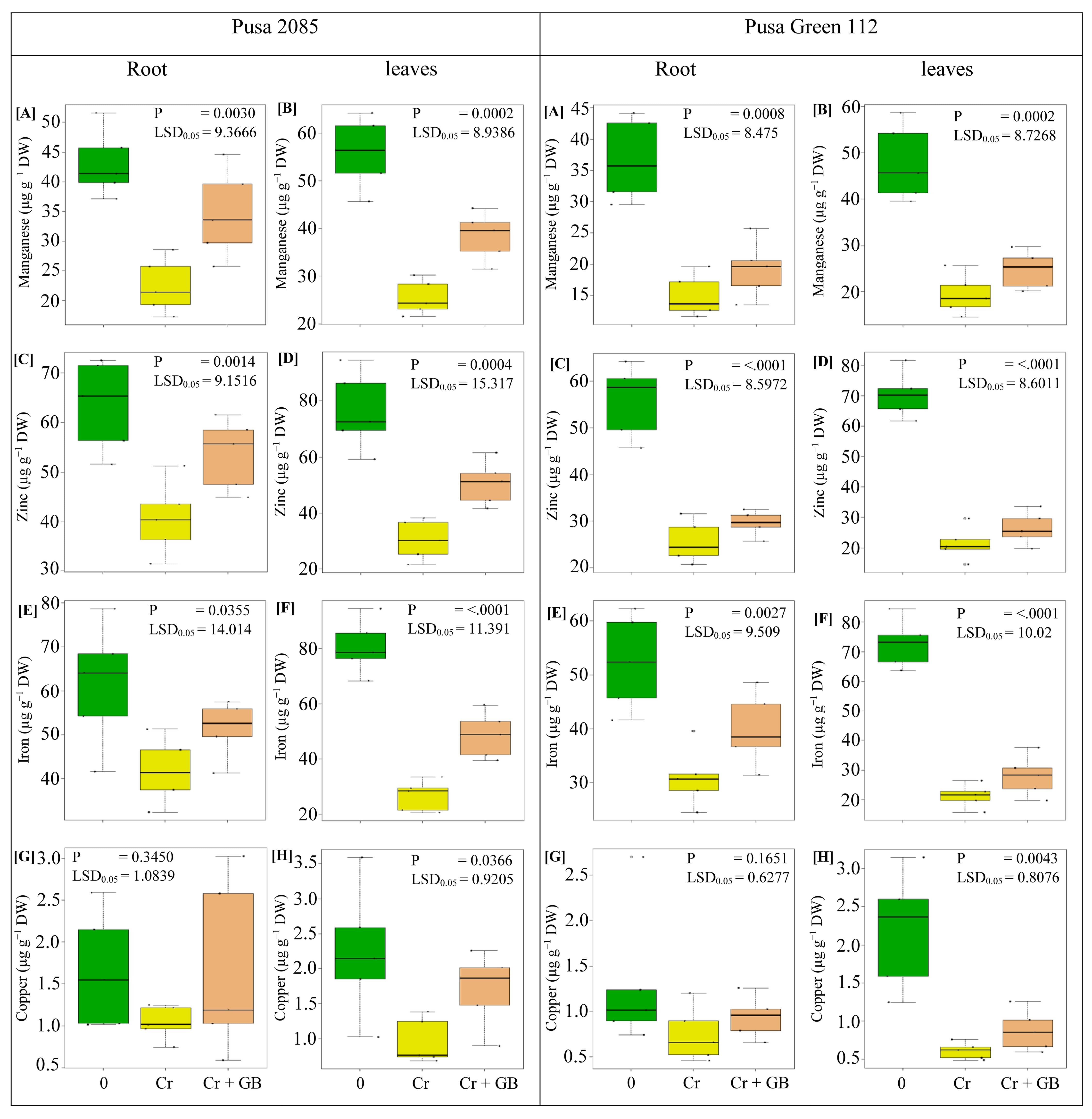

Supplement: Supplementary file 3 — Supplementary Figure S3. [file 41598_2022_11869_MOESM3_ESM.jpg]
