# Supplementary material for: Glycine betaine modulates chromium (VI)-induced morpho-physiological and biochemical responses to mitigate chromium toxicity in chickpea (Cicer arietinum L.) cultivars
Source: Sci Rep. 2022 May 14;12:8005. doi: 10.1038/s41598-022-11869-3 (PMC9107477; doi:10.1038/s41598-022-11869-3)
Supplement: Supplementary file 4 — Supplementary Table S1. [file 41598_2022_11869_MOESM4_ESM.docx]

**Additional File 4. Table S1.** The initial chemical proprieties of the loamy soil are listed.

| Basic organic properties | loam sand |
| --- | --- |
| Sand (%) | 56.00 |
| Silt (%) | 20.00 |
| Clay (%) | 24.00 |
| Organic matter (%) | 0.37 |
| Phosphorous (P) mg kg ⁻^1^ | 21.40 |
| Potassium (K) mg kg ⁻^1^ | 176.0 |
| Iron (Fe) mg kg ⁻^1^ | 1.24 |
| Nitrogen (N) mg kg ⁻^1^ | 6.54 |
| Electrical conductivity (dsm⁻^1^) | 2.60 |
| Calcium carbonate (CaCO_3_) mg kg ⁻^1^ | 1.54 |
| Copper (Cu) mg kg ⁻^1^ | 0.002 |
| Manganese (Mn) mg kg ⁻^1^ | 0.045 |
| Zinc (Zn) mg kg ⁻^1^ | 0.07 |
| Chromium (Cr) | BDL |
| Lead (Pb) | BDL |
| Cadmium (Cd) mg kg ⁻^1^ | 0.001 |
| pH | 7.31 |
| Aluminum (Al) mg kg ⁻^1^ | 0.004 |

Note: - BDL- Below Detection Limit
