# Supplementary material for: Glycine betaine modulates chromium (VI)-induced morpho-physiological and biochemical responses to mitigate chromium toxicity in chickpea (Cicer arietinum L.) cultivars
Source: Sci Rep. 2022 May 14;12:8005. doi: 10.1038/s41598-022-11869-3 (PMC9107477; doi:10.1038/s41598-022-11869-3)
Supplement: Supplementary file 5 — Supplementary Legends. [file 41598_2022_11869_MOESM5_ESM.docx]

**Additional File 1. Fig. S1.** Effects of different stress levels of Glycine Betaine (GB) and Chromium (Cr) at 0 and 120 μM Cr and 120 μM Cr + 100 mM (GB) on (**A**) Chl-a levels (mg g^−1^), (**B**) Chl-b levels and (**C**) Total chl levels in the leaves of chickpea cultivars, Pusa 2085 and Pusa Green 112 under pot conditions at 42 days after sowing. The generated data values are shown as the mean of five replicates ± SE. The error bars indicated by the different letters are significant differences, as tested by the least significant difference test (p < 0.05).

**Additional File 2. Fig. S2.** Box plot showing the levels of macronutrients (N, P, K, Ca, and Mg) in the root and leaf tissues of both chickpea cultivars (Pusa 2085 and Pusa Green 112) grown in the presence of 0 and 120 µM Cr and 120 µM Cr + 100 mM glycine betaine (GB) in pots at 42 days after sowing. Least significant differences (LSD_0.05_) were calculated for all the treatments using SAS 9. 4 software.

**Additional File 3. Fig. S3.** Box plot showing the levels of microelements (Mn, Zn, Fe, and Cu) in the root and leaf tissues of both chickpea cultivars (Pusa 2085 and Pusa Green 112) grown in the presence of 0 and 120 µM Cr and 120 µM Cr + 100 mM glycine betaine (GB) in pots at 42 days after sowing. Least significant differences (LSD_0.05_) were calculated for all the treatments using SAS 9.4 software.

**Additional File 4. Table S1.** The initial chemical proprieties of the loamy soil.
